# Prevalence of Varicose Veins Among Teaching Professionals and Their Impact on Quality of Life and Job Performance: A Cross-Sectional Study

**DOI:** 10.3390/healthcare13233041

**Published:** 2025-11-25

**Authors:** Safaa M. Elkholi, Danah Alotaibi, Reem Alrashdi, Reema Bin Subeh, Hajer Aljudeie, Rema Aljabr, Eman M. Mortada, Reem M. Alwhaibi

**Affiliations:** 1 Department of Rehabilitation Sciences, College of Health and Rehabilitation Sciences, Princess Nourah bint Abdulrahman University, P.O. Box 84428, Riyadh 11671, Saudi Arabia; 2 Family and Community Medicine Department, College of Medicine, Princess Nourah bint Abdulrahman University, P.O. Box 84428, Riyadh 11671, Saudi Arabia

**Keywords:** varicose veins, teaching professionals, quality of life, work performance, occupational health, static posture, ergonomic risk, cross-sectional study

## Abstract

**Background**: Varicose veins (VVs) are a chronic venous condition that can negatively affect mobility, psychosocial health, and occupational function, especially in professions involving prolonged standing or sitting. Teaching professionals are particularly at risk due to static postural demands and limited workplace ergonomic support. **Objective**: This study aimed to estimate the prevalence of VVs among teaching professionals in Saudi Arabia and assess their impact on quality of life (QoL) and job performance. **Methods**: A cross-sectional study was conducted employing a stratified convenience sampling strategy among 400 school and university educators across different regions of Saudi Arabia. Data collection took place over four months, from mid-January to end-April 2025. Data were collected through a validated self-administered online questionnaire comprising demographic information and three standardized tools: The Arabic version of the Aberdeen Varicose Vein Questionnaire (AVVQ), the World Health Organization Quality of Life–BREF (WHOQOL-BREF), and Individual Work Performance Questionnaire (IWPQ). Descriptive statistics, *t*-tests, ANOVA, chi-square, and correlation were used for analysis. Ethical approval for this study was obtained from the Institutional Review Board of Princess Nourah bint Abdulrahman University (IRB Log Number: 25-0008). **Results**: The prevalence of VVs was 18.8%. Male gender, prolonged static postures, and obesity were significantly associated with higher VV rates (*p* < 0.05). Logistic regression identified gender as the only independent predictor of VV presence (*p* < 0.001). Participants with VVs reported significantly lower QoL across all WHOQOL domains and reduced work performance scores. Two-way ANOVA showed a significant main effect of gender on work performance (*p* = 0.002), while VV status and occupation showed no significant interaction effects. VV severity was negatively correlated with job performance (r = −0.138, *p* = 0.006), while QoL positively correlated with performance (r = 0.149, *p* = 0.003). University faculty demonstrated significantly higher VV severity than schoolteachers (*p* = 0.013). **Conclusions**: It is concluded that the prevalence of varicose veins among teaching professionals in Saudi Arabia is associated with significantly lower quality of life and reduced work performance, highlighting the occupational impact of the condition. Preventive workplace interventions and further longitudinal research are recommended to confirm and expand these findings.

## 1. Introduction

Varicose veins (VVs) are a common form of chronic venous disorder characterized by dilated, tortuous, and elongated superficial veins of the lower limbs. They develop due to venous valve incompetence that leads to retrograde blood flow, increased venous pressure, and progressive vein wall dilation. The condition represents both a clinical and a socioeconomic concern, as it is associated with chronic pain, swelling, and impaired mobility that may restrict daily activities and work performance [1]. Globally, the prevalence of VVs ranges from 10% to 30%, with higher rates reported among women, older adults, and individuals exposed to occupations requiring prolonged static postures [2,3]. Although often perceived as a cosmetic problem, accumulating evidence demonstrates that VVs substantially affect physical functioning, psychological well-being, and quality of life (QoL) [4].

Both modifiable and non-modifiable risk factors contribute to the development of VVs. These include age, sex, obesity, smoking, pregnancy, hormonal therapy, genetic predisposition, and occupational exposure to prolonged standing or sitting [4]. Physiologically, static postures reduce calf-muscle pump activity, elevate venous pressure, and may eventually lead to valvular insufficiency [5]. In occupational settings, such mechanical strain is accentuated among workers who stand for long periods or remain seated for extended hours without sufficient movement, thereby increasing venous stasis and vascular load [6,7].

Teaching professionals represent one of the occupational groups at greatest risk for venous disorders because their work demands alternate between long standing during instruction and prolonged sitting for grading and administrative duties. In Saudi Arabia, teachers in schools often spend several continuous hours standing and moving among students, whereas university faculty members typically engage in extended sedentary activities such as lecturing from seated positions, preparing examinations, or conducting research. These contrasting postural patterns may expose educators at both levels to distinctive venous risk profiles [3,6]. Emerging occupational health studies also suggest that university faculty may experience greater venous strain than schoolteachers due to longer uninterrupted periods of sitting and reduced daily mobility, potentially leading to higher VV severity in this subgroup [6,7]. Despite this, the occupational burden of VVs among educators remains under-investigated, and available studies have been limited to single regions, small samples, or specific educational levels, making it difficult to generalize findings across the Saudi academic workforce [3,6,8].

Current evidence rarely integrates vascular health outcomes with validated assessments of QoL and job performance, leaving important gaps in understanding the functional consequences of VVs for professional productivity and well-being. In the Saudi context, where the teaching profession constitutes a large and sedentary segment of the workforce, identifying these associations is essential for developing effective preventive and ergonomic strategies. A comprehensive evaluation that includes both school and university educators is particularly warranted to capture the diversity of occupational exposures across educational settings [6,7,8].

Accordingly, this study aimed to (1) estimate the prevalence of varicose veins among teaching professionals in Saudi Arabia and identify associated occupational risk factors, and (2) examine the relationship between varicose veins, quality of life, and individual work performance. It was hypothesized that VVs prevalence among educators would exceed that of the general population due to their occupational posture demands, and that affected educators would demonstrate lower QoL and reduced work performance, with differences expected between school and university settings. By addressing this gap, the study provides evidence that may guide targeted interventions and policy initiatives to enhance occupational vascular health and productivity among educators in Saudi Arabia.

## 2. Methodology

### 2.1. Study Design

This study adopted a cross-sectional survey design to evaluate the prevalence and severity of VVs and their associated impact on quality of life and job performance among teaching professionals (schoolteachers and university faculty members) in Saudi Arabia. This design is appropriate for estimating population-level prevalence and identifying relationships between risk factors and health outcomes at a single point in time.

The research was conducted over a four-month period from mid-January to the end of April 2025, allowing sufficient time for participant recruitment, online questionnaire distribution, and data monitoring. Ethical approval for the study was obtained from the Institutional Review Board of Princess Nourah bint Abdulrahman University (IRB Log Number: 25-0008), confirming that the study met international ethical standards for research involving human participants. Participants were provided with detailed information about the study and gave informed electronic consent prior to enrollment.

Using the PECO framework, the structured research question guiding this study was: Among teaching professionals in Saudi Arabia (Population), how do occupational exposures such as prolonged standing or prolonged sitting (Exposure), compared with educators without these exposure characteristics (Comparison), relate to the prevalence and severity of varicose veins and their impact on quality of life and work performance (Outcomes)?

### 2.2. Participants

The target population included both schoolteachers and university faculty members working across Saudi Arabia, as both groups face considerable occupational strain due to prolonged standing while teaching and extended sitting during administrative duties.

Participants were eligible for inclusion if they were educators aged 25 years or older, currently employed in Saudi Arabia, had at least two years of teaching experience, and predominantly stood or sat for extended periods during their workday. Individuals were excluded if they reported severe, disabling, or work-preventing chronic diseases, cardiovascular conditions, a history of lower extremity injury, arterial disease, pregnancy, or previous surgical or medical interventions related to venous disorders.

For the subgroup analyses, participants were classified as “VV-positive” only if they had a prior clinical diagnosis of varicose veins confirmed by a healthcare professional. This distinction was made because the Aberdeen Varicose Vein Questionnaire (AVVQ) is a patient-reported outcome measure (PROM) and does not independently establish a clinical diagnosis. This strategy ensured that the VV subgroup was accurately defined while minimizing confounding factors that could influence the development or progression of varicose veins.

**Figure 1:**
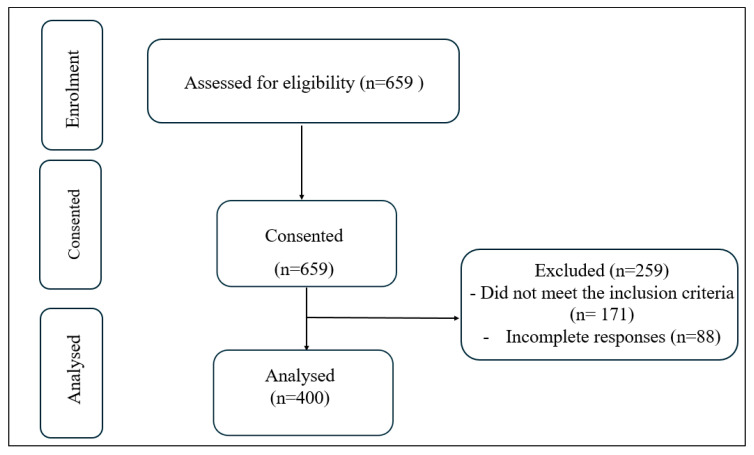
Flow chart of participants’ recruitment.

Figure 1 illustrates the recruitment and selection process for participants. A total of 659 individuals were initially assessed for eligibility, and all provided informed consent. Following screening, 171 individuals were excluded for not meeting the predefined inclusion criteria, which required active employment as teaching professionals in Saudi Arabia, with at least two years of teaching experience, and regular engagement in prolonged sitting or standing. Participants were also excluded for reporting lower extremity injury, arterial disease, pregnancy, or prior surgical/medical interventions related to venous conditions. An additional 88 individuals were excluded due to incomplete survey responses. Thus, a final sample of 400 eligible participants with complete data was included in the analysis.

### 2.3. Sample Size Calculation

According to the most recent national statistics, the total number of school and university teaching professionals in Saudi Arabia was estimated at 518,000 in 2024, comprising 454,600 schoolteachers and 63,400 university faculty members, as reported by the Ministry of Education.

The required sample size was calculated using OpenEpi version 3.01, assuming a 95% confidence interval, a design effect of 1.0, an expected frequency (prevalence) of 20% based on previous Saudi studies, and a margin of error of 5%. The calculated minimum sample size was 385 participants. To enable subgroup comparisons between schoolteachers and university faculty, the final target sample was increased to 500 to maintain adequate statistical power.

### 2.4. Sampling Technique

A stratified random sampling approach was not feasible due to logistical constraints; therefore, a stratified purposive (non-probability) convenience sampling method was adopted. Stratification was performed according to teaching level (school or university), gender, subject area (sciences, humanities, or health-related), and region (urban vs. rural). Within each stratum, participants who met inclusion criteria were invited to participate during the recruitment period.

To minimize selection bias, recruitment was conducted through official institutional email networks and professional educators’ associations rather than open social media links. Each potential participant received the same standardized invitation text and consent form to ensure uniformity of information.

Although convenience sampling may introduce selection bias and limit generalizability, this approach was justified by the large geographic spread of the teaching population and the need for rapid, accessible recruitment of busy professionals. The potential bias associated with this method is acknowledged in the study’s limitations section.

### 2.5. Data Collection Tool

Data were collected using a validated, structured online questionnaire developed on Google Forms. To minimize duplicate submissions, the Google Form prevented multiple entries from the same device by activating Google’s response-limit settings and by reviewing the dataset for repeated IP/device identifiers. No duplicate entries were detected. The instrument assessed four domains: demographic and occupational data, VVs symptoms, quality of life, and work performance.

1. **Demographic and Occupational Information**

Collected variables included age, gender, marital status, educational level, teaching level (school or university), job title, subject area, years of experience, and daily duration of standing or sitting.

2. **Aberdeen Varicose Veins Questionnaire (AVVQ)—Arabic Version**

The AVVQ is a 13-item patient-reported outcome measure designed to assess the physical, psychosocial, and functional impact of varicose veins, rather than to diagnose them. It captures symptom severity, cosmetic concerns, and functional limitations, generating scores from 0 to 100, with higher scores reflecting greater disease impact. The Arabic version used in this study has been validated and demonstrates high reliability (α = 0.87, *p* < 0.01) [9,10].

3. **Individual Work Performance Questionnaire (IWPQ)**

This 18-item tool measures job performance across three domains: task performance, contextual performance, and counterproductive work behavior. Each item is rated on a 5-point Likert scale (1 = seldom to 5 = always). Scores are calculated by averaging responses within each domain. This instrument has been widely used in occupational health research for its robust psychometric properties. The IWPQ has demonstrated strong internal consistency with Cronbach’s alpha values ranging from 0.79 to 0.89, and high test–retest reliability (intraclass correlation coefficients up to 0.98), supporting its reliability. Additionally, studies confirm its construct validity for assessing individual work performance across occupational settings [11].

4. **World Health Organization Quality of Life-BREF (WHOQOL-BREF)—Arabic Version**

This internationally standardized 26-item questionnaire evaluates four key domains: Physical Health, Psychological Well-being, Social Relationships, and Environmental Factors. Domain scores are calculated, transformed to a 0–100 scale, and interpreted such that higher scores reflect better quality of life. The Arabic version has demonstrated acceptable reliability and validity in various populations [12,13].

### 2.6. Validation and Pilot Testing

All standardized instruments used in this study (AVVQ, IWPQ, WHOQOL-BREF) were administered in their Arabic-language versions. The IWPQ underwent a comprehensive linguistic validation process, including forward and backward translation by bilingual experts, followed by a panel review to ensure semantic and conceptual equivalence. A pilot study was conducted with 20 participants from both school and university levels to assess clarity, length, and comprehension. Based on participant feedback, minor wording changes were made to reduce ambiguity in the occupational exposure questions (e.g., clearer definitions of “prolonged standing” and “sedentary sitting”), and question order was optimized for logical flow. The final questionnaire required approximately 12–15 min to complete and was available via both desktop and mobile devices for accessibility.

### 2.7. Procedures

Potential participants were contacted through official institutional email channels. Each invitation included a brief explanation of the study objectives, voluntary participation, and confidentiality measures, along with a secure link to the questionnaire. Electronic informed consent was obtained before survey access. Participants could withdraw at any time without penalty.

To ensure data integrity, incomplete responses were automatically excluded, and preliminary checks were performed to identify potential duplicate entries (e.g., highly similar timestamps, repeated demographic patterns, and identical answer sequences). Although IP-based filtering was applied as an additional precaution, we acknowledge that respondents could theoretically access the questionnaire from different devices or networks. However, the dataset was thoroughly screened for unusual repetition patterns or anomalies, and no indications of duplicate submissions were detected. All data were anonymized and securely stored on password-protected drives accessible only to the principal investigator and authorized team members, in full compliance with institutional ethical standards and international best practices for data privacy.

### 2.8. Data Analysis

All collected data were coded, entered, and analyzed using the Statistical Package for the Social Sciences (SPSS), version 30.0.

Descriptive statistics were used to summarize participants’ demographic, occupational, and health-related characteristics and measure the prevalence of VVs. Frequencies and percentages were reported for categorical variables, while means and standard deviations were computed for continuous variables.

To determine the association between the VVs and associated risk factors, cross-tabulations were performed. Chi-square tests were used to assess associations between categorical variables such as gender, work habits (e.g., prolonged standing or sitting), medical history, and lifestyle behaviors (e.g., exercise, smoking, obesity). Statistical significance was set at *p* < 0.05. Normality of continuous dependent variables was assessed using the Kolmogorov–Smirnov test. Variables that met the criteria for normal distribution were analyzed using independent samples *t*-tests and one-way ANOVA, which were applied to compare the mean scores of HRQOL-BREFF, its four domains, and work performance between participants with and without varicose veins, as well as across subgroups such as gender, job title, and type of teaching institution (school vs. university). Moreover, Mann–Whitney U test was used to compare median scores for quality-of-life ratings and participants’ self-reported satisfaction with their overall health.

To further investigate the combined influence of varicose vein status with other variables, two-way ANOVA models were conducted to assess both main effects and interaction effects between varicose vein status and gender and between varicose vein status and occupational category on work-performance scores.

Spearman correlation analysis was conducted to examine the relationships between the prevalence of varicose veins, quality of life (WHOQOL-BREF), and job performance (IWPQ). These correlations helped to assess the direction and strength of the associations among key study variables.

In addition, a binary logistic regression model was performed to evaluate the predictive value of gender, age, and occupation on the presence of varicose veins (yes/no), given the binary nature of the outcome. Model fit statistics, odds ratios, and confidence intervals were reported.

Effect sizes were interpreted where applicable, and all assumptions for parametric tests were checked. Non-parametric alternatives were not required, as normality was reasonably met by the primary outcomes. Subgroup analysis was also conducted to compare university faculty and schoolteachers in terms of varicose vein prevalence, quality of life, and work performance.

These comparisons aimed to explore occupational-level differences in outcomes and assess the potential role of work setting in influencing vascular health.

## 3. Results

### 3.1. Demographic and Work-Related Characteristics of Teaching Staff

As shown in Table 1, 82.3% of participants were female and 75.0% were married. The most common age bracket was 35–39 years (37.8%). Nearly half held a bachelor’s degree (48.0%), and 35.8% held a doctorate. Teachers constituted 40.0% of the sample, followed by assistant professors (20.0%), professors (14.8%), and associate professors (11.5%). More than half (54.5%) had ≥10 years of experience. Regarding daily exposure, 41.0% reported standing 4–6 h, 23.0% ≥ 6 h; 46.0% reported sitting 1–3 h, and 19.3% ≥ 6 h.

### 3.2. Health and Work Conditions of Teaching Professionals

Table 2 summarizes participants’ health and occupational characteristics. Most respondents reported prolonged standing (85.0%) and prolonged sitting (52.5%) during work; 93.5% had ≥2 years of teaching experience, and 29.0% reported comorbid chronic conditions (e.g., diabetes, hypertension). A total of 18.8% (75/400) reported a clinician-confirmed diagnosis of varicose veins (VVs), corresponding to a prevalence of 18.8% (95% CI: 15.2% to 22.9%). Thirteen percent reported prior surgical/medical interventions for VVs.

### 3.3. Prevalence of Risk Factors for VVs Among Participants

Table 3 shows risk-factor distributions. Positive family history was reported by 43.3%. Physical inactivity was common: 45.5% exercised “rarely,” and 6.5% exercised daily. Overweight/obesity was present in 39.8%, hormonal therapy use in 34.3%, and chronic constipation in 27.3%. Smoking prevalence was 8.8%. Rheumatoid arthritis (8.8%), kidney disease (3.8%), and prior DVT (4.8%) were infrequent.

### 3.4. Factors Associated with Varicose Veins

Table 4 presents a bivariate analysis of factors associated with the presence of VVs among teaching professionals. Significant associations were identified for gender, standing and sitting durations, prolonged sitting behavior, and overweight/obesity status.

Male participants were significantly more likely to have VVs compared to females (91.2% vs. 8.8%; *p* = 0.000). Work posture patterns were also strongly associated with varicose vein prevalence. Participants who stood for 4–6 h daily reported the highest prevalence (32.4%), while those who stood more than six hours had lower rates (26.4%), possibly due to adaptation or selection bias. Sitting for 4–6 h was significantly associated with VVs (28.2%; *p* = 0.000), and participants who reported sitting for prolonged periods were more likely to have VVs (59.7% vs. 44.0%; *p* = 0.002).

Obesity was another significant predictor, with 46.8% of participants with VVs reporting being overweight or obese, compared to 31.5% of those without (*p* = 0.002). In contrast, age group, exercise frequency, and smoking were not significantly associated with varicose vein status in this sample (*p* > 0.05).

### Determinants of Varicose Vein Prevalence: Bivariate and Logistic Regression Analyses

The distribution of varicose vein prevalence by gender, age, and occupation is presented in Table 5. A significant association was observed between gender and varicose vein prevalence (χ^2^ = 25.785, *p* < 0.001), with female participants showing a higher prevalence (26.8% with varicose veins) compared to males (59.9% with varicose veins). This finding indicates that females are more susceptible to varicose veins, likely due to hormonal factors, pregnancy, and occupational postures.

No significant associations were found between age and varicose vein prevalence (χ^2^ = 6.264, *p* = 0.281), although slightly higher prevalence rates were observed in participants aged 35–60 years. Similarly, occupation was not significantly associated with varicose vein prevalence (χ^2^ = 0.485, *p* = 0.486), with university staff (55.4% with varicose veins) and school teachers (51.9% with varicose veins) exhibiting comparable rates. These results suggest that, within this sample, age and professional category do not substantially influence varicose vein occurrence.

A binary logistic regression model was conducted to assess the predictive value of gender, age, and occupation on the presence of varicose veins (Table 6). The model was statistically significant (Omnibus Test of Model Coefficients: χ^2^ = 29.924, df = 3, *p* < 0.001), indicating that the set of predictors collectively distinguished between participants with and without varicose veins. The model explained 7–10% of the variance (Cox & Snell R^2^ = 0.072; Nagelkerke R^2^ = 0.096) and correctly classified 62.3% of cases, performing better for participants with varicose veins (91.2%) than for those without (28.3%).

Among the predictors, gender was a significant determinant (B = 1.468, *p* < 0.001; Exp(B) = 4.342, 95% CI: 2.438–7.734), indicating that females were more than four times as likely as males to have varicose veins. Age demonstrated a positive but non-significant trend (B = 0.139, *p* = 0.088; Exp(B) = 1.150), whereas occupation did not significantly predict varicose vein prevalence (B = –0.092, *p* = 0.671; Exp(B) = 0.912). The correlation matrix confirmed that multicollinearity was not a concern among the predictors.

Overall, both analyses consistently highlight gender as the primary factor associated with varicose vein prevalence, whereas age and occupation did not show significant effects. These findings suggest that preventive measures and workplace interventions should particularly target high-risk groups, notably females, rather than focusing solely on age or occupational category.

### 3.5. Impact of VVs on Quality of Life

Table 7 reveals the differences in the quality of life of the study groups using Independent *T*-test with significant differences existing in WHOHRQOL and all domains of QOL with the mean scores which were significantly higher in participants without Varicose veins (*p*s < 0.05).

Similarly, on using Mann–Whitney test, significant differences exist in the median scores between the study groups regarding their rating of quality of life and level of satisfaction their health (U = 1.2813, Z = −6.36, and U = 1.2114, Z = −6.96, *p* < 0.001, respectively).

### 3.6. Impact of VVs on Work Performance

Table 8 presents the mean work-performance scores according to gender, occupational category, and the presence or absence of varicose veins. The analysis revealed a significant main effect of gender on work performance, with female participants demonstrating generally higher performance scores than their male counterparts across most occupational positions. For instance, among participants without varicose veins, females reported higher mean scores in the Associate Professor (3.54 ± 0.47) and Assistant Professor (3.59 ± 0.54) categories compared to males (3.12 ± 0.64 and 3.14 ± 0.44, respectively). A similar trend was observed among those with varicose veins, where females showed comparatively higher scores in several roles, such as Assistant Professor (3.77 ± 0.00) and Teaching Assistant (3.60 ± 0.19).

In contrast, the results indicated no significant effect of occupation on work performance, as the differences observed among the various job titles did not reach statistical significance. Although descriptive means varied slightly—particularly within the university staff categories—these variations did not represent meaningful differences in performance across occupational groups.

Similarly, no significant effect of the presence or absence of varicose veins was observed. While participants with varicose veins tended to exhibit marginally lower performance scores (e.g., male lecturers: 2.76 ± 0.51) compared to those without the condition (3.25 ± 0.35), these differences were not statistically significant. Furthermore, the interaction effects between gender, occupation, and varicose vein status were also non-significant, indicating that the combined influence of these variables did not contribute meaningfully to variations in performance.

Overall, the findings suggest that gender is the only variable exerting a significant influence on work performance, whereas occupational role and varicose vein status—whether individually or interactively—do not significantly affect performance levels.

### 3.7. Relationship Between VVs and QoL/Work Performance

Table 9 presents the Spearmen correlation coefficients examining the relationships among varicose vein severity (measured by the AVVQ), quality of life (measured by WHOQOL-BREF), and job performance (measured by the IWPQ). A significant negative correlation was found between varicose vein severity and job performance (r^s^ = −0.164 **, *p* = 0.006), indicating that higher symptom burden was associated with slightly lower perceived work performance. Another significant negative correlation between varicose vein severity and quality of life (r^s^ = −0.298, *p* = 0.001). A significant strong positive correlation was observed between quality of life and job performance (r^s^ = 0.971 **, *p* <0.001).

### 3.8. The Impact of VVs on Quality of Life and Job Performance Among Teaching Professionals

Table 10 compares varicose vein severity, quality of life, and job performance between university and school teaching professionals. A statistically significant difference was observed in varicose vein severity, with university faculty reporting higher mean AVVQ scores (M = 11.04, SD = 11.32) compared to schoolteachers (M = 8.43, SD = 8.50; *p* = 0.013).

Quality of life scores were nearly identical between the two groups (University: M = 3.20, SD = 0.70; School: M = 3.22, SD = 0.72; *p* = 0.314). Similarly, work performance scores showed no significant difference between university (M = 3.08, SD = 0.55) and school staff (M = 3.15, SD = 0.49; *p* = 0.196).

## 4. Discussion

This study explored the prevalence, severity, and occupational implications of varicose veins (VVs) among teaching professionals in Saudi Arabia. The findings highlight a multifactorial interplay of occupational exposure, lifestyle behaviors, and physiological predisposition contributing to the disease burden within this population. Notably, the results revealed that VV severity differed according to gender, occupational environment, and role-specific work patterns, emphasizing the need to consider contextual occupational factors when addressing venous health among educators.

The observation that male participants demonstrated higher VV severity than females contrasts with most epidemiological literature, which commonly identifies women as the higher-risk group due to hormonal influences, pregnancy, and contraceptive use [14,15]. This reversal could reflect occupational exposure differences; male faculty, particularly in university settings, may spend more time in static seated or standing postures during lectures or research supervision. Cultural or behavioral variations in symptom reporting may also play a role, as female educators could be more proactive in adopting preventive measures such as compression hosiery or leg elevation, thereby mitigating symptom progression [16,17,18]. Such findings suggest that gendered patterns of occupational exposure and health-seeking behavior warrant deeper investigation in future research.

The institutional context also emerged as a key determinant of VV severity. University faculty reported higher symptom scores than schoolteachers, consistent with occupational health evidence linking sedentary work and diminished calf-muscle pump activity to venous stasis [19,20]. The work routines of university staff often involve long hours of sitting for grading, administrative duties, and research, which reduces venous return. In contrast, schoolteachers are generally more mobile within classrooms, an activity pattern that intermittently stimulates venous flow and may reduce venous pressure accumulation. This differentiation underscores that the physical design of work, not merely professional category, significantly affects venous health outcomes.

Differences across academic ranks also reflected varied exposure demands. Lecturers and assistant professors, whose responsibilities combine teaching, research, and administrative work, experienced the highest VV severity. These mixed-role positions entail prolonged desk work interspersed with infrequent movement breaks, resulting in greater venous strain. Such role-specific exposure is similar to that seen in occupations with limited mobility, including nursing and surgical work [2,4]. Recognizing these job-embedded risks enables more tailored ergonomic and preventive strategies targeting specific academic subgroups rather than uniform institutional policies.

Consistent with global evidence, several modifiable risk factors, including prolonged sitting and standing, excess body weight, and family history, were significantly associated with VV presence and severity. Overweight individuals are predisposed to venous congestion due to elevated intra-abdominal pressure and mechanical compression on the lower limb veins, which hinders efficient venous return [21,22]. Similarly, prolonged immobility, whether sitting or standing, leads to sustained venous hypertension, a central mechanism in the pathogenesis of chronic venous disease [23,24]. These findings align with WHO recommendations that emphasize interrupting sedentary time with light-intensity movement to improve vascular and metabolic health [25].

The multivariable logistic regression findings further refined these associations by showing that gender was the only independent predictor of VV presence, whereas age and occupational category did not significantly contribute to the model. Female educators had more than four-fold higher odds of having VVs compared with males, even after adjusting for age and occupation, which is consistent with large population-based studies reporting a higher burden of chronic venous disease among women [7,21,22].

Taken together with the bivariate analyses, these results indicate that although certain work-related and lifestyle factors cluster with VV occurrence, underlying sex-related susceptibility remains a dominant determinant of disease presence in this population.

A distinctive pattern emerged when considering quality of life (QoL) and job performance. Varicose vein severity showed modest but significant negative correlations with both QoL and work performance, while between-group comparisons demonstrated that participants with VVs had significantly lower WHOQOL-BREF scores than those without the condition across all domains. This pattern suggests that the mere presence of VVs is sufficient to exert a clinically relevant impact on QoL, whereas incremental increases in severity within the range observed in this sample are associated with only small additional decrements. This can be interpreted through both psychometric and psychosocial lenses. Generic QoL tools such as WHOQOL-BREF are less sensitive than disease-specific instruments (e.g., Aberdeen Varicose Veins Questionnaire (AVVQ), Chronic Venous Insufficiency Questionnaire (CIVIQ)) to localized venous symptoms and early functional limitations, which may attenuate the strength of severity–QoL correlations despite clear differences between affected and unaffected groups [26,27]. Moreover, educators may adapt their behavior by using coping strategies (e.g., leg elevation, stretching, compression garments), preserving general well-being even as work-related functioning declines. The professional satisfaction and autonomy inherent to teaching may also buffer perceived QoL, a phenomenon described as “occupational identity resilience” in similar health conditions [7,23]. In contrast, job performance measures, focused on concentration, stamina, and physical tolerance, are more sensitive to mild physical impairments. Consequently, our findings support the interpretation that VVs negatively affect both QoL and work performance, with performance measures capturing early functional impacts that may not yet be fully reflected in global QoL scores.

These results are broadly in line with international evidence linking chronic venous disease to impaired QoL and functional limitations. Studies using AVVQ, CIVIQ, and other disease-specific Patient-Reported Outcome Measures have consistently shown that higher venous disease severity is associated with poorer physical functioning, pain, and reduced SF-36 or SF-12 scores, as well as improvement in QoL after VV treatment [28,29,30,31]. Reviews of chronic venous disease emphasize not only the symptomatic burden but also its impact on work productivity and daily activities, including loss of working days and reduced performance at work [32,33]. Evidence from teachers and other standing professions (e.g., nurses, hospital staff) similarly indicates that varicose veins and chronic venous symptoms are common and negatively influence comfort, mobility, and perceived ability to perform job duties [3,34,35,36]. Our findings extend this literature by quantifying both QoL and work performance in school and university educators using validated instruments, reinforcing the view that VVs in teaching professionals should be regarded as an occupational health concern rather than a purely cosmetic issue.

The two-way ANOVA models provided additional details regarding work performance by showing that gender was the only factor with a significant main effect, whereas VV status, occupational category, and their interaction terms did not reach statistical significance. This suggests that the modest associations between VV severity and performance observed in correlation analyses may be partly explained by gender-related differences in work roles, activity patterns, or coping strategies. It is also possible that many educators with VVs in our sample had mild-to-moderate disease that allowed them to maintain near-normal performance scores on the IWPQ, consistent with reports that decrements in productivity become more pronounced in more advanced stages of chronic venous disease or in physically demanding occupations such as nursing [32,35]. These findings highlight the need for future research to disentangle biological, occupational, and gendered behavioral factors that shape how venous disease translates into perceived work performance.

Overall, these findings affirm that VVs should be viewed not as a cosmetic or isolated vascular disorder but as a relevant occupational health issue within the education sector. Teaching professionals face prolonged static postures and restricted mobility that increase venous pressure and fatigue, ultimately diminishing work efficiency. Incorporating simple preventive measures, such as scheduled movement breaks, posture variation, ergonomic seating, and workplace education, could reduce symptom severity and enhance productivity. These recommendations are consistent with international occupational health literature supporting the introduction of low-cost workplace interventions to reduce venous disease risk and sedentary behavior [24,37]. Future research should build on these results using longitudinal designs and validated diagnostic criteria to confirm causality and inform evidence-based workplace health policies.

### 4.1. Limitations

This study has several methodological and contextual limitations that should be considered when interpreting the findings. First, the cross-sectional design prevents the establishment of causal relationships between occupational exposure and VV outcomes; longitudinal follow-up is needed to confirm temporal directionality. Second, data were self-reported, which introduces the potential for recall bias and misclassification, particularly for clinical variables such as VV diagnosis and symptom severity. Although validated instruments were used, the absence of ultrasound or other imaging confirmation may limit diagnostic accuracy. Third, non-probability online sampling may have led to selection bias, as participants experiencing VV symptoms could have been more motivated to respond, potentially inflating prevalence and severity estimates. Fourth, while the study adjusted for key confounders such as obesity and work exposure, unmeasured variables (e.g., genetic predisposition, physical activity intensity) may still have influenced outcomes. Finally, the study’s focus on school and university educators restricts generalizability to other occupational groups with different postural demands. These limitations collectively suggest caution when extrapolating the results, yet they do not diminish the study’s contribution to understanding VV risk in academic professions.

### 4.2. Contributions to Knowledge and Study Strengths

This study provides several important contributions to the current understanding of VVs among occupational groups, particularly within educational settings. First, it is one of the few studies in Saudi Arabia, and the first to our knowledge, to examine VVs among both schoolteachers and university faculty using a large sample size drawn from multiple regions. Previous studies have been limited to either small samples or single occupational levels, restricting generalizability and providing an incomplete picture of venous health among educators [3,6,8]. By including diverse teaching roles and institutions, the present study offers a more comprehensive representation of occupational exposure patterns and their vascular implications.

Second, the integration of validated standardized tools (AVVQ, WHOQOL-BREF, and IWPQ) allows for a multidimensional assessment of venous disease, quality of life, and work performance. This is a notable advancement over prior research, which often assessed VVs without linking them to functional or occupational outcomes. By correlating VV severity with both QoL and work performance, this study provides novel evidence of the occupational and psychosocial consequences of venous disease, an area that remains underexplored in the literature [26,27].

Third, the study contributes new insights related to gender-specific occupational patterns. While international literature consistently identifies females as having higher VV risk due to hormonal and physiological factors [14,15], our findings reveal a reverse pattern within the Saudi educator population, suggesting that occupational exposure, movement patterns, and behavioral factors may outweigh biological risk in certain work environments. This observation highlights the need for more detailed analyses in venous health research and opens new perspectives for targeted interventions in male-dominated academic roles.

Finally, the study provides valuable evidence supporting the classification of VVs as an occupational health concern rather than merely a cosmetic issue, reinforcing the importance of ergonomic strategies and workplace mobility policies. These findings align with international occupational health guidelines urging reductions in sedentary behavior and the implementation of preventive measures in the workplace [24,28].

Overall, the study advances knowledge by broadening the occupational lens through which VVs are understood, integrating functional outcomes, and identifying critical gender and role-based patterns that have not been previously documented in the regional or global literature.

### 4.3. Future Work

Future research should pursue longitudinal and clinically validated investigations to clarify the causal pathways linking occupational exposure and venous outcomes. Integrating objective diagnostic methods such as duplex ultrasound will strengthen diagnostic precision and enable the development of predictive models based on exposure duration, posture, and body composition. Experimental or quasi-experimental studies are recommended to evaluate the efficacy of ergonomic interventions, including sit-stand desks, anti-fatigue mats, and scheduled mobility breaks, in reducing venous symptoms and improving work performance. Additionally, future studies should examine gender-specific occupational exposures to better explain the higher VV severity observed among male educators and to design interventions tailored to these patterns. Implementing workplace wellness programs that promote physical activity, weight management, and early detection may further reduce VV prevalence and related occupational strain. Such evidence-based approaches can guide institutional health policies that enhance both staff well-being and productivity in educational settings.

## 5. Conclusions

The present study provides a comprehensive assessment of the prevalence, severity, and occupational correlates of varicose veins among teaching professionals in Saudi Arabia. The findings reveal that VVs represent a significant yet under-recognized occupational concern within academic environments, with higher severity observed among university staff and male educators—patterns that diverge from global gender trends. This divergence likely reflects differences in occupational exposure, posture demands, and self-management behaviors. Furthermore, the observed association between VV severity and diminished work performance underscores the tangible functional consequences of venous insufficiency in professions characterized by static postures and limited mobility.

The absence of a corresponding decline in overall quality of life suggests that educators may adapt psychologically and behaviorally to physical symptoms through resilience, intrinsic job satisfaction, and compensatory strategies. However, such adaptation does not eliminate the physical burden of the condition and may mask underlying occupational risk. Recognizing varicose veins as an occupational health issue rather than a purely cosmetic or age-related problem is essential for improving both individual well-being and institutional productivity. Targeted preventive measures addressing posture, physical activity, and weight management could mitigate VV risk, enhance teaching performance across educational levels, and help reduce long-term vascular complications.

## Figures and Tables

**Table 1:** Demographic and Work-Related Characteristics of Teaching Staff (n = 400).

Variable	Response	Frequency	Percent
Gender	Female	329	82.3
	Male	71	17.8
Age groups (y)	<35	50	12.5
	35–39	151	37.8
	40–44	53	13.3
	45–49	92	23.0
	50–60	44	11.0
	Over 65	10	2.50
Marital Status	Single	61	15.3
	Married	300	75
	Divorced	30	7.5
	Separated	9	2.3
Education Level	Bachelor’s degree	192	48.0
	High school	3	0.8
	Doctorate (PhD)	143	35.8
	Fellowship	1	0.3
	Master’s degree	57	14.3
Job Title	Professor	59	14.8
	Assistant Professor	80	20.0
	Associate Professor	46	11.5
	Lecturer	33	8.3
	Teacher	160	40.0
	Teaching Assistant	22	5.5
Experience (years)	≤1	29	7.3
	1–5	72	18.0
	6–10	81	20.3
	≥10	218	54.5
Teaching/Standing per Day (hours)	≤1	14	3.5
	1–3	130	32.5
	4–6	164	41.0
	≥6	92	23.0
Sitting per Day (hours)	≤1	76	19.0
	1–3	184	46.0
	4–6	63	15.8
	≥6	77	19.3

**Table 2:** Health and Occupational Characteristics of the Study Sample (n = 400).

Variable	Response	n	Percent
Teaching Status	Yes	400	100
Prolonged Standing at Work	No	60	15
	Yes	340	85
Prolonged Sitting at Work	No	190	47.5
	Yes	210	52.5
≥2 Years Teaching Experience	No	26	6.5
	Yes	374	93.5
Chronic Disease or Cardiovascular Conditions	No	275	68.8
	Yes	125	31.3
History of Lower Limb Injury/Arterial Disease	No	331	82.8
	Yes	69	17.3
(For female participants) pregnant	No	304	92.4
	Yes	25	7.6
Surgical/Medical Treatment for VVs	No	348	87
	Yes	52	13
Confirmed Diagnosis of VVs	No	325	81.3
	Yes	75	18.8
Comorbid Chronic Conditions (e.g., diabetes, hypertension)	No	284	71
	Yes	116	29

**Table 3:** Distribution of Risk Factors Associated with VVs among Teaching Professionals (n = 400).

Variable	Response	Frequency	Percent
Family History of VVs	No	227	56.8
	Yes	173	43.3
Exercise Frequency	2–3 times per week	86	21.5
	Never	45	11.3
	Weekly	61	15.3
	Rarely	182	45.5
	Daily	26	6.5
Smoking Status	No	365	91.3
	Yes	35	8.8
Deep Vein Thrombosis (DVT)	No	381	95.3
	Yes	19	4.8
Kidney Disease	No	385	96.3
	Yes	15	3.8
Rheumatoid Arthritis	No	365	91.3
	Yes	35	8.8
History of Lower Limb Injury	No	326	81.5
	Yes	74	18.5
Overweight or Obesity	No	241	60.3
	Yes	159	39.8
Use of Hormonal Therapy	No	175	43.8
	Not applicable	88	22.0
	Yes	137	34.3
Tight Clothing Use	No	334	83.5
	Yes	66	16.5
High Heel Use	No	223	55.8
	Not applicable	74	18.5
	Yes	103	25.8
Chronic Constipation	No	291	72.8
	Yes	109	27.3

**Table 4:** Factors Associated with the Prevalence of VVs among Teaching Professionals (n = 400).

Variables		Varicose Veins				
		Without Varicose Veins		With Varicose Veins		*p*-Value
		Frequency	%	Frequency	%	
Gender	Female	52	28.3	19	8.8	0.000 *
	Male	132	71.7	197	91.2	
Age	25–30 years	30	16.3	20	9.3	0.281
	35–40 years	69	37.5	82	38.0	
	40–45 years	23	12.5	30	13.9	
	45–50 years	37	20.1	55	25.5	
	55–60 years	19	10.3	25	11.6	
	Over 65 years	6	3.3	4	1.9	
Teaching/Standing per Day (hours)	≤1	8	4.3	6	2.8	0.000 *
	1–3	81	44.0	83	38.4	
	4–6	22	12.0	70	32.4	
	≥6	73	39.7	57	26.4	
Sitting per Day (hours)	≤1	37	20.1	39	18.1	0.000 *
	1–3	27	14.7	36	16.7	
	4–6	16	8.7%	61	28.2	
	≥6	104	56.5	80	37.0	
Sit for long periods during working hours	No	103	56.0	87	40.3	0.002 *
	Yes	81	44.0	129	59.7%	
Exercise Frequency	2–3 times per week	22	12.0	23	10.6	0.053
	Never	92	50.0	90	41.7	
	Weekly	27	14.7	34	15.7	
	Rarely	38	20.7	48	22.2	
	Daily	5	2.7	21	9.7	
Smoking Status	No	168	91.3	197	91.2	0.972
	Yes	16	8.7	19	8.8	
Overweight or Obesity	No	126	68.5	115	53.2	0.002 *
	Yes	58	31.5	101	46.8	

* Significant at a significance level of 0.05.

**Table 5:** Distribution of Varicose Vein Prevalence According to Gender, Age, and Occupation.

Variables	Categories	Presence of Varicose Veins		Chi-Square	*p*-Value
		Without Varicose Veins	With Varicose Veins		
		n (%)	n (%)		
Gender	Female	52 (73.2)	19 (26.8)	25.785	0.000 *
	Male	132 (40.1)	197 (59.9)		
Age	25–30 years	30 (60.0)	20 (40.0)	6.264	0.281
	35–40 years	69 (45.7)	82 (54.3)		
	40–45 years	23 (43.4)	30 (56.6)		
	45–50 years	37 (40.2)	55 (59.8)		
	55–60 years	19 (43.2)	25 (56.8)		
	Over 65 years	6 (60.0)	4 (40.0)		
Occupation	University Staff	107 (44.6)	133 (55.4)	0.485	0.486
	School Teacher	77 (48.1)	83 (51.9)		

* significant at 0.01.

**Table 6:** Logistic Regression Analysis Predicting the Presence of Varicose Veins Based on Gender, Age, and Occupation.

Variables	B	S.E.	Wald	df	Sig.	Exp(B)	95% C.I. for EXP(B)	
							Lower	Upper
Gender	1.468	0.295	24.847	1	0.000	4.342	2.438	7.734
Age	0.139	0.082	2.911	1	0.088	1.150	0.979	1.349
Occupation	−0.092	0.217	0.181	1	0.671	0.912	0.596	1.396
Constant	−2.798	0.719	15.136	1	0.000	0.061		

**Table 7:** Comparison of Quality of Life Between Participants with and without VVs (n = 400).

	Study Groups				
Variables	Without VV (No. = 184)	With VV (No. = 216)	Overall (No. = 400)	Test	*p*-Value
Overall QOL Mdn (IQR)	1(2)	2(1)	1(1)	1.2813 ^U^ (z = −6.36)	<0.001 *
Overall health satisfaction Mdn (IQR)	0.5(2)	2(2)	2(2)	1.2114 ^U^ (z = −6.96)	<0.001 *
Physical health M ± SD	16.5 ± 2.065	15.49 ± 2.12	15.89 ± 2.14	2.933 ^T^	0.004 *
Psychological health M ± SD	13.83 ± 2.59	12.8 ± 2.40	13.35 ± 2.53	3.522 ^T^	<0.001 *
Social relations M ± SD	16.21 ± 2.75	15.28 ± 2.61	15.71 ± 2.71	3.462 ^T^	0.001 *
Environment M ± SD	12.92 ± 2.33	11.99 ± 2.15	12.41 ± 2.28	4.164 ^T^	<0.001 *
WHOQOL-BREF M ± SD	64.85 ± 8.96	60.04 ± 8.28	62.19 ± 8.89	3.174 ^T^	0.002 *

VV: Varicose Veins, U = Mann–Whitney test; T = independent T test; M: mean; SD: standard deviation; WHOQOL-BREF = World Health Organization Quality of Life-Brief, Mdn = median, IQR = iInterquartile Range, * Significant difference (*p* ≤ 0.05).

**Table 8:** Descriptive Statistics for Job Performance by Gender, Varicose Vein Status, and Occupation.

Presence/Absence of Varicose Veins ^ns^	Gender *	Occupation ^ns^					
		University Staff					School Teacher
		Professor	Associate Professor	Assistant Professor	Lecturer	Teaching Assistant	
Without varicose veins	Female	3.04 ± 0.91	3.54 ± 0.47	3.59 ± 0.54	3.52 ± 0.79	3.05 ± 0.65	3.36 ± 0.61
	Male	3.16 ± 0.42	3.12 ± 0.64	3.14 ± 0.44	3.25 ± 0.35	3.16 ± 0.41	3.12 ± 0.48
With varicose veins	Female	2.92 ± 0.6	3.22 ± 0.36	3.77 ± 0.00	3.15 ± 0.00	3.60 ± 0.19	3.22 ± 0.31
	Male	2.86 ± 0.69	3.03 ± 0.52	3.07 ± 0.46	2.76 ± 0.51	2.73 ± 0.38	3.12 ± 0.46

^ns^ means that there is no significant effect of the interacted variables on the work performance. * refers to significant single effect of the variable on the work performance (*p*-value = 0.002).

**Table 9:** Correlation between Varicose Veins Severity, Quality of Life, and Job Performance among Teaching Professionals (n = 400).

		Prevalence of Varicose Veins	Quality of Life	Job Performance
**Prevalence of Varicose Veins**	Spearman Correlation	1.000	−0.298 **	−0.164 **
	Sig. (2-tailed)	.	0.001	0.001
**Quality of life**	Spearman Correlation	−0.298 **	1.000	0.971 **
	Sig. (2-tailed)	0.001	.	0.000
**Job performance**	Spearman Correlation	−0.164 **	0.971 **	1.000
	Sig. (2-tailed)	0.001	0.000	.

** Correlation is significant at the 0.01 level (2-tailed).

**Table 10:** Comparison of Varicose Vein Severity, Quality of Life, and Job Performance Between University and School Teaching Professionals (n = 400).

Variables	University Staff		School Teacher		*p*-Value
	Mean	SD	Mean	SD	
**Severity level of VVs**	11.04	11.32	8.43	8.50	0.013 *
**Quality of life**	3.20	0.70	3.22	0.72	0.314
**Job performance**	3.08	0.55	3.15	0.49	0.196

* Significant difference (*p* ≤ 0.05).

## Data Availability

The data supporting the findings of this study are available from the corresponding author upon reasonable request. The data are not publicly available due to ethical restrictions and the confidentiality agreement approved by the Institutional Review Board of Princess Nourah bint Abdulrahman University.
